# An EST resource for tilapia based on 17 normalized libraries and assembly of 116,899 sequence tags

**DOI:** 10.1186/1471-2164-11-278

**Published:** 2010-04-30

**Authors:** Bo-Young Lee, Aimee E Howe, Matthew A Conte, Helena D'Cotta, Elodie Pepey, Jean-Francois Baroiller, Federica di Palma, Karen L Carleton, Thomas D Kocher

**Affiliations:** 1 Department of Biology, University of Maryland, College Park, Maryland 20742, USA; 2 CIRAD-PERSYST, Aquaculture Research Unit, TA B-20/A, Campus International de Baillarguet, 34398 Montpellier cedex 5, France; 3 Broad Institute, Cambridge, Massachusetts 02142, USA

## Abstract

**Background:**

Large collections of expressed sequence tags (ESTs) are a fundamental resource for analysis of gene expression and annotation of genome sequences. We generated 116,899 ESTs from 17 normalized and two non-normalized cDNA libraries representing 16 tissues from tilapia, a cichlid fish widely used in aquaculture and biological research.

**Results:**

The ESTs were assembled into 20,190 contigs and 36,028 singletons for a total of 56,218 unique sequences and a total assembled length of 35,168,415 bp. Over the whole project, a unique sequence was discovered for every 2.079 sequence reads. 17,722 (31.5%) of these unique sequences had significant BLAST hits (e-value < 10^-10^) to the UniProt database.

**Conclusion:**

Normalization of the cDNA pools with double-stranded nuclease allowed us to efficiently sequence a large collection of ESTs. These sequences are an important resource for studies of gene expression, comparative mapping and annotation of the forthcoming tilapia genome sequence.

## Background

Tilapia is the common name for a group of 40-50 nominal species of cichlid fishes (Order Perciformes) native to Africa and the Middle East. Tilapias have been an important aquaculture species for thousands of years. Sometimes called the 'aquatic chicken', tilapia are sturdy and adaptable fish, and are now cultured in more than 100 countries in Asia and the Americas [1]. Tilapias are a cornerstone of future aquaculture production, and genetic improvement of this species is needed to increase growth rate and improve disease resistance. Cichlid fishes are also important models for research on vertebrate physiology, behavior, and evolutionary biology. Because of their close evolutionary relationship, genetic resources developed for tilapia are also useful for studying the extraordinary radiation of haplochromine cichlids in the lakes of East Africa [2].

Genetic resources for tilapia are relatively well developed, and include a microsatellite-based genetic map [3] and a physical map based on BAC fingerprints [4]. In contrast, EST resources for these species are limited. Modest EST projects have been published for some haplochromine cichlids [5-7]. Although several labs have constructed cDNA libraries for tilapia, and a few thousand ESTs have been characterized [8-10], until now large-scale sequencing of ESTs has not been pursued in tilapia.

The applications of a high-quality EST resource are manifold. Many researchers are interested in constructing microarrays to study changes in gene expression during development [11] and in response to environmental stressors including handling [12], temperature [13], salinity [14], disease [15,16] and environmental contaminants [17-19]. ESTs will provide an important resource for annotating the forthcoming tilapia genome sequence. A major effort is needed to enlarge the database of cichlid ESTs to facilitate analysis of gene expression and contribute to genome annotation. Here we describe the sequencing of almost 117,000 ESTs from highly normalized libraries constructed from 16 tissues of the Nile tilapia, *Oreochromis niloticus*.

## Results

### cDNA libraries and EST sequences

We constructed a total of two non-normalized and 17 normalized libraries from 16 tissues or developmental time points (Table 1). Three normalized libraries captured gene expression during early development (pooled whole animals at 0-4 days, 5-15 days, and 16-40 days post fertilization). Normalized libraries were also constructed for brain, heart, kidney, liver, olfactory epithelium, skin, skeletal muscle, spleen and stomach. Normalized libraries were constructed separately for adult ovary and testis, along with a normalized mixed library of differentiating ovary and testis from 14 - 56 days post-fertilization. For gill and retina, paired libraries (non-normalized and normalized) were compared.

**Table 1:** cDNA libraries from *Oreochromis niloticus.*

Lib name	Tissue	Description	Reads
Br3	Brain	normalized	11,520
D0-4	Whole embryos	normalized	9,120
D5-15	Whole larvae	normalized	9,216
D16-40	Whole juveniles	normalized	9,216
Gi1	Gill	non-normalized	6,144
Gi2	Gill	normalized	4,992
GOHD	Mixed gonad	normalized	5,123
Ht2	Heart	normalized	4,992
Ki3	Kidney	normalized	6,144
Li6	Liver	normalized	4,992
Oe1	Olfactory epithelium	normalized	9,216
Ov1	Ovary	normalized	9,216
Re3	Retina	non-normalized	3,840
Re4	Retina	normalized	15,027
Sk1	Skin	normalized	4,992
Sm1	Skeletal muscle	normalized	4,992
Sp1	Spleen	normalized	4,992
St1	Stomach	normalized	4,992
Te2	Testis	normalized	8,928

**Figure 1:**
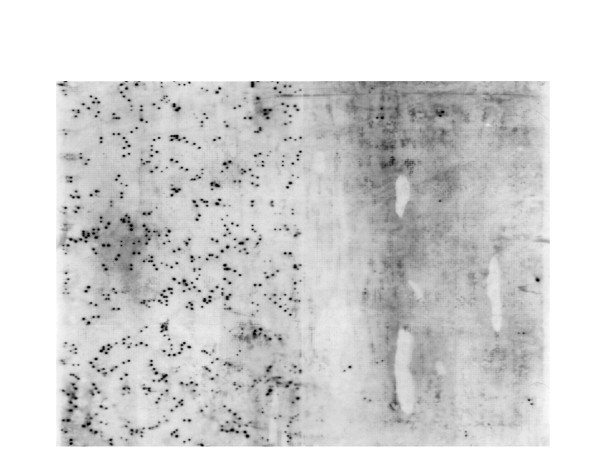
**A single high density filter of retinal cDNA libraries hybridized with a rhodopsin probe**. The left side of the filter contains ~9,000 clones from an un-normalized library. The right half of the filter contains ~9,000 clones from the normalized library. Normalization has greatly reduced the redundancy of the library.

The normalization procedure was highly effective. Figure 1 presents Southern blots for non-normalized and normalized retinal libraries probed for rhodopsin. Each panel contains approximately 9,000 clones. The non-normalized panel shows approximately 360 clones positive for rhodopsin. The normalized panel shows only about 9 positive clones. For this gene then, the number of duplicate clones has been reduced approximately 40-fold.

We sequenced approximately 96 clones from each library to check the size and quality of the inserts. We then submitted approximately 5,000 clones from each library for high-throughput sequencing. After analysis of these sequences, we selected some libraries for additional sequencing. We obtained at least partial sequence data from 137,654 clones. The number of sequences obtained from each library is shown in Table 1. The sequences were then passed through the EST2uni analysis pipeline [20]. This pipeline includes a preprocessing step that includes vector trimming and masking of repetitive and low complexity sequences. The total number of reads passing the trimming step was 116,899 (84.9%).

### Clustering of tilapia ESTs

**Figure 2:**
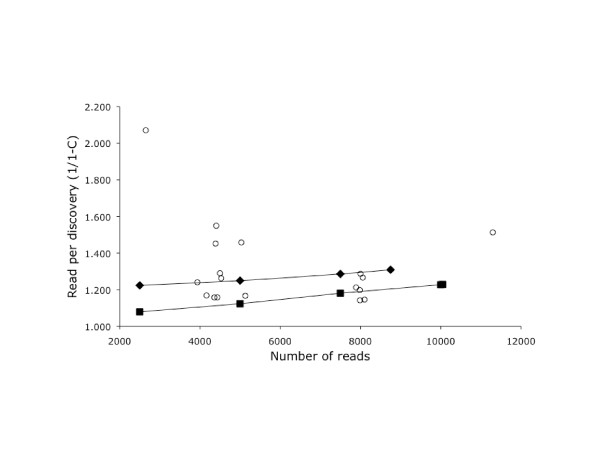
**The redundancy of each library at different depths of sequencing**. The x-axis is the number of sequence reads from each library. The y-axis indicates the number reads required to discover a sequence that does not cluster with the existing sequences for that library. Results after each round of sequence are shown for Br3 (squares) and Ret4 (diamonds). Other libraries are shown with circles. The point in the upper left is the non-normalized Ret3 library.

We performed separate Cap3 assemblies to assess the rate of sequence discovery for each library (Table 2). After sequencing 5,000 clones from each library, the rate of discovery ranged from 1.1 to 1.6 reads/discovery (Figure 2). This quantification allowed us to select the least redundant libraries for further sequencing.

**Table 2:** Clustering statistics for each library.

Lib name	HQ ESTs	Contigs	Singletons	Total	Coverage	Discovery
Br3	10,051	1,245	6,935	8,180	0.1862	1.229
D0-4	7,891	991	5,517	6,508	0.1753	1.213
D5-15	8,001	1,045	5,171	6,216	0.2231	1.287
D16-40	8,101	773	6,294	7,067	0.1276	1.146
Gi1	5,032	576	2,875	3,451	0.3142	1.458
Gi2	4,164	357	3,206	3,563	0.1443	1.169
GOHD	3,936	367	2,806	3,173	0.1939	1.240
Ht2	4,529	633	2,954	3,587	0.2080	1.263
Ki3	5,131	526	3,871	4,397	0.1431	1.167
Li6	4,360	459	3,306	3,765	0.1365	1.158
Oe1	7,983	797	5,866	6,663	0.1654	1.198
Ov1	7,988	772	6,221	6,993	0.1246	1.142
Re3*	2,650	374	906	1,280	0.5170	2.070
Re4*	11,298	2,162	5,306	7,468	0.3390	1.309
Sk1	4,406	714	2,130	2,844	0.3545	1.549
Sm1	4,434	465	3,363	3,828	0.1367	1.158
Sp1	4,389	964	2,059	3,023	0.3112	1.452
St1	4,498	695	2,791	3,486	0.2250	1.290
Te2	8,057	1,134	5,228	6,362	0.2104	1.266

* Includes forward and reverse reads for some clones

As expected, the number of reads required to discover a new sequence increased over time. Figure 2 plots the discovery statistic for the brain3 and retina4 libraries at different levels of sequence coverage. Both libraries showed a gradual increase in the number of reads required to discover a new sequence.

### Final assembly

**Figure 3:**
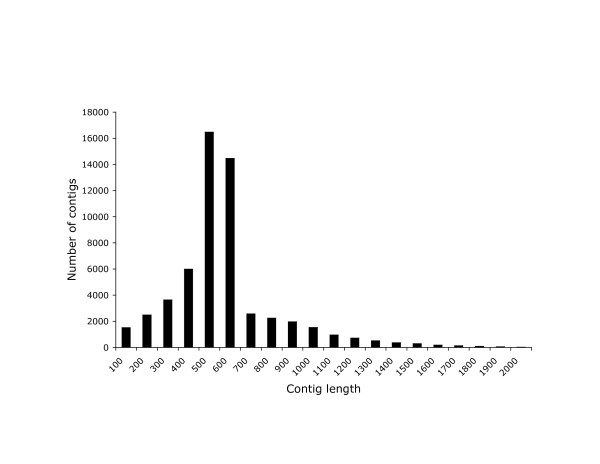
**Size distribution of the contigs in 100 bp bins**.

After sequencing of all the libraries was complete, we analyzed all of the sequences together to produce a single assembly consisting of 56,218 unigenes averaging 625.6 bp in length, for a total assembled length of 35,168,415 bp. The distribution of contig lengths is shown in Figure 3.

The average number of ESTs per unique sequence (contigs + singletons) was 2.1. The average number of ESTs per contig was 4.0, and the maximum number of ESTs in a contig was 404. The nine contigs with more than 80 sequences each were mostly genes that we expect to be highly expressed, including hemoglobin, rhodopsin, parvalbumin, MHC class I, and mitochondrial cytochrome c oxidase subunits 1, 2 and 3. Two of these exceptionally large contigs appeared to be artifacts representing assembly of repetitive sequences.

**Figure 4:**
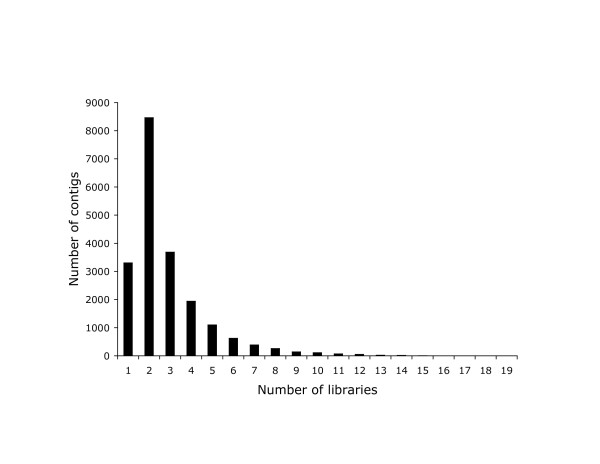
**Number of contigs by the number of libraries in which that sequence is expressed**.

Figure 4 shows the number of libraries that contributed sequences to each contig. The modal value is two, and very few contigs contain sequences from more than five libraries. This is partly explained by the small number of sequences contributing to each contig, but also indicative of the low redundancy across libraries.

### Sequence similarity and functional annotation

**Figure 5:**
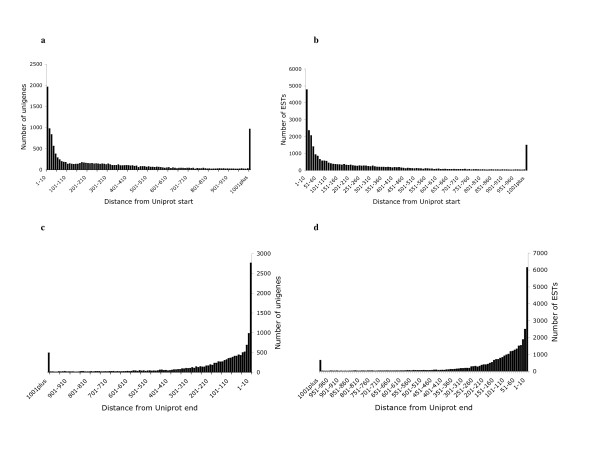
**Distribution of sequence starts and stops on Uniprot entries**. a, c - distributions for unigenes. b, d - distributions for unassembled ESTs.

41,129 (35%) of the unassembled ESTs and 17,722 (32%) of the assembled unigenes found a significant (e^-10^) blast match in the UniProt database. We calculated the fraction of ESTs and Unigenes that were complete on the 5' and 3' ends by scoring the number of sequences that matched to within 10 amino acids of the 5' and 3' ends of each UniProt entry (Figure 5). This proportion varied with the length of the UniProt entry (Table 3). Sixty-five percent of the ESTs matching UniProts <250aa were complete on the 5' end. For UniProts between 251 and 500 amino acids this fraction dropped to 36%. Similarly, 68% of the unigenes matching UniProts <250aa were complete on the 5' end, and the fraction dropped to 36% for UniProts between 251 and 500 amino acids. Overall 13.9% of the ESTs and 13.1% of the unigenes were complete on both the 5' and 3' ends. 17,505 (31%) of the unigenes were mapped to candidate Gene Ontology (GO) terms. Following the application of the annotation rules, 12,792 (22.8%) of the unigenes were annotated with GO terms. The proportion of unigenes annotated with GO terms can be seen for each of the three GO functional categories (biological process, molecular function, and cellular component) in Additional file 1: Figure S1. 10,527 (18.7%) of the unigenes had a single-directional best hit to the KEGG pathway database.

**Table 3:** Distribution of ESTs and Unigenes on Uniprot entries. The criteria for completeness of the EST was whether it reached within 10 amino acids of the end of the Uniprot entry. Values indicate the proportions within each size class.

ESTs	< 250aa	251-500aa	501-750aa	751-1000aa	> 1000aa
5' & 3'	0.31	0.00	0.00	0.00	0.00
5' only	0.34	0.36	0.13	0.10	0.07
3' only	0.20	0.21	0.27	0.27	0.23
incomplete	0.15	0.43	0.60	0.63	0.70
total	18,369	13,510	4,432	1,892	2,926
				sum	41,129
**Unigenes**					

5' & 3'	0.38	0.04	0.00	0.00	0.00
5' only	0.30	0.32	0.15	0.11	0.08
3' only	0.18	0.24	0.29	0.28	0.24
incomplete	0.14	0.40	0.56	0.61	0.68
total	5,421	6,277	2,740	1,238	2,046
				sum	17,722

### Microsatellites and SNPs

We identified 1,108 microsatellites in the assembled sequences. These include 592 dinucleotide, 319 trinucleotide and 197 tetranucleotide repeat loci. The most abundant repeats were AC dinucleotides (511 instances) followed by AAT trinucleotides (99 instances). Most of the microsatellites identified were in the 3'UTR (46%) or the 5'UTR (30%). Only 24% were found within the open reading frame.

The EST2uni pipeline identified 48,309 candidate SNPs in the assembled contigs. For 2,542 of these candidate SNPs each of the alternative bases was represented at least twice. Of these, 1329 (52%) represented transition substitutions and 1213 (48%) represented transversions.

## Discussion

### Effectiveness of normalization

Normalization of copy number during library construction produces a more even distribution of clone frequencies, increasing the rate of gene discovery [21]. When very large numbers of ESTs are needed, serial-subtractive hybridization is an effective, but technically demanding, method for removing duplicate clones [22,23]. An alternative is cherry-picking of unique clones identified by filter hybridization [24].

We normalized the cDNA population before cloning using a duplex-specific nuclease from the Kamchatka crab [25]. This enzyme can be used to degrade rapidly hybridizing components of a cDNA mixture, reducing the representation of highly expressed genes. This method proved highly effective, reducing the representation of the more abundant transcripts by 40-fold. We further increased our chances of detecting rare transcripts by constructing libraries from a large number of different tissues and developmental stages. The overall rate of gene discovery for this project compares favorably with projects of similar scope.

To further study the effectiveness of normalization, we used BLAST to screen the normalized retinal library for genes expected from rod and cone phototransduction. We identified rhodopsin as well as 6 of the 7 cone opsins in these libraries (all but RH2B). We further identified numerous genes from the rod phototransduction pathway, including all three subunits of the G protein (GNAT1, GNB1, GNGT1), phosphodiesterase subunits (PDE6a, PDE6b, PDE6g), the sodium/calcium exchanger (NCKX1) and G protein kinase (GRK1). For the cone pathway, we were able to find arrestin (Arr3), but did not identify other phototransduction proteins. In most vertebrates, rod photoreceptors are more prevalent than cone photoreceptors. In fish the ratio or rods to cones is typically 50:1 [26,27]. The expression of the various phototransduction genes is less than opsin by factors of 10 (G protein) to 100 (PDE) or more [28]. Our normalization scheme reduced the representation of the most highly expressed genes by a factor of 40. If expression of genes in the cone pathway is 50× less frequent than that of the rod, and if the components of the rest of the phototransduction pathway are 10× less frequent, it is perhaps not surprising that we only observed cone opsins and cone arrestin from the cone pathway. While this confirms the effectiveness of our normalization methods, it suggests that additional genes would be discovered if we continued to sequence these highly normalized libraries.

### Anchors for comparative mapping

Microsatellites associated with genes provide an efficient means to construct comparative maps among species [29]. In channel catfish, as many as 12% of ESTs contain microsatellite sequences [30]. Using roughly the same search parameters, we found microsatellites in only 870 of 56,218 (1.5%) of tilapia unigenes. Nevertheless, these 870 microsatellites provide a useful starting point for comparative mapping [31]. The subset of high confidence SNPs is another starting point for constructing a comparative genetic map of tilapia with other fish species.

## Conclusion

Our project has significantly enhanced the EST resources available for cichlid fishes. While the total number of ESTs for tilapia still lags behind that for other fish species, our use of multiple highly normalized libraries has contributed to a high level of gene discovery. The number of UniProt hits for our EST collection rivals that for other model fish species [32,33]. The total length of the assembled transcriptome (35 Mb) is similar to the total length of the annotated regions of the Tetraodon (33.9 Mb) and Takifugu (29.1 Mb) genomes [34]. This annotated collection of clustered ESTs will be a key resource for annotation of cichlid genome sequences for studies of cichlid physiology, development and evolution.

## Methods

### Construction of the 19 tilapia cDNA libraries

#### cDNA library construction

Total and poly(A)+ RNA were extracted from homogenized tilapia tissues using the RNAeasy and Oligotex mRNA kits (Qiagen) respectively. Full-length cDNAs were prepared using the SMART technology [35]. First strand and second strand cDNA was synthesized from 0.5 to 1 μg of mRNA using the "Creator SMART cDNA Library Construction Kit" (Clontech) according to the manufacturers instructions. The terminal transferase activity of MMLV reverse transcriptase was used to add untemplated C residues at the 3' end of the first strand cDNA. The second strand is primed with a SMART oligo sequence ending with three G residues (dAAGCAGTGGTATCAACGCAGAGTGGCCATTACGGCCGGG) which pair with the added C's. The SMART anchor oligo, together with a modified oligo(dT) primer (dATTCTAGAGGCCGAGGCGGCCGACATG(T)_30_NN) or (dAAGCAGTGGTATCAACGCAGAGTGGCCGAGGCGGCC(T)_20_VN) was then used to amplify double-stranded cDNA by long distance (LD) PCR. Only those cDNAs with the SMART anchor at the 5' end should be amplified.

#### DSN normalization of SMART amplified cDNA

Normalization of the SMART amplified double-stranded cDNA was achieved using the Kamchatka crab duplex-specific nuclease [28]. In brief, double-stranded cDNA was denatured and allowed to reassociate under second order reaction kinetics. The ds cDNA fraction formed by abundant transcripts during reassociation is then degraded by DSN which displays a strong preference for cleaving ds DNA in both DNA-DNA and DNA-RNA hybrids compared to ssDNA or RNA. The resulting equalized single-stranded fraction was then amplified using long-distance PCR. Enzyme dilution was optimized and number of PCR cycles was minimized for each tissue in order to preserve the quality of the equalized cDNA.

#### Ligation and cloning

The incorporation of asymmetrical SfI sites in the multiple cloning site of the pDNR-LIB cloning vector allowed directional cloning of SMART SfI restricted dscDNA. Since SfI sites are extremely rare in vertebrate DNA, almost all SMART cDNAs remain intact after SfI digestion. Double stranded SfI restricted cDNA was size fractionated using CHROMA SPIN-400 columns (Clonetech, Mountain View, CA). Sixteen fractions were collected and analyzed by agarose gel electrophoresis. Those fractions containing the highest molecular weight cDNAs (longer than 0.5 kb) were cloned into the pDNR-lib plasmid vector.

#### EST sequencing

The majority of the single pass 5' Sanger sequence reads were contracted to Agencourt Bioscience (Beverly MA). Bacterial clones were inoculated and grown overnight at 37°C. High-copy plasmid templates were purified using a streamlined SprintPrep™ SPRI^® ^protocol [36,37]. This procedure harvests plasmid DNA directly from lysed bacterial cultures by trapping both plasmid and genomic DNA to the functionalized bead particles and selectively eluting only the plasmid DNA. DNA templates were sequenced in 384-well format using BigDye^® ^Version 3.1 reactions and purified using Agencourt's CleanSeq^® ^dye-terminator removal kit before separation on ABI3730xl instruments (Applied Biosystems, Foster City, CA). All reads were processed using Phred base calling software. A passing read was defined as an average high quality PHRED score of 20 or higher for at least 100 bases.

For the normalized and non-normalized retinal libraries, both 5' and 3' reads were contracted to SymBio Corporation (Menlo Park CA). Clones were amplified by Templiphi (GE Healthcare Life Sciences, Piscataway, NJ) and then sequenced in 384 well format on a MegaBACE sequencer (Molecular Dynamics now GE Healthcare). Base calling and quality scores were generated with PHRED.

### Data extraction and quality filtering

#### Trimming

Components of the EST2uni analysis pipeline were compiled and run on an Intel Mac OS X server. Default parameters provided by each of the EST2uni components were used except where noted henceforth. Base calling was performed by Phred [38], known vector trimming was performed by Lucy [39], repeat masking was performed with RepeatMasker, version open-3.2.7, with database version 20090120 [40]. Unknown vector and low complexity masking was performed by SeqClean. Default settings of the vector and quality-trimming program Lucy were modified in order to adequately trim our reads. The *max_avg_error *defined in the Lucy manual corresponds to the quality score of a base and was lowered from 0.02 (Q17) to 0.001 (Q30) for the -*bracket *setting which controls the removal of low quality bases from both ends of a sequence. The -*window *setting uses a sliding window algorithm to check if regions of a specified window size are within a *max_avg_error*. Multiple windows can be specified and the default window of length 10 and *max_avg_error *of 0.3 was changed to a *max_avg_error *of 0.063 (while keeping the length of 10) to increase the stringency of quality trimming.

#### Redundancy

After each round of sequencing we performed a statistical analysis to estimate the complexity and coverage of each library. The statistical methodology for this analysis was developed by Susko and Roger [40]. For a given number of reads from a single library, we can estimate the depth of sequencing coverage of the library as:

where n_1 _is the number of genes that appear just once in the sample and n is the total number of reads. From this estimate of coverage we can also calculate the number of additional reads required to discover a new gene:

### Assembly

CAP3 [41] was used to perform assembly of the processed ESTs. The -*p *setting, representing overlap percent identity cut-off, was set to 90. The -*d *setting, specifying max qscore sum at differences, was set to 110. The *est_clustering.pm *script in the EST2uni package had to be slightly modified to parse the assembly output correctly.

### Annotation

#### BLAST

Assembled ESTs were annotated by running BLASTX against UniProt release 15.2 with an E value cutoff of e^-10 ^to designate similar sequences and e^-25 ^to designate highly similar sequences.

#### Position of ESTs along Uniprot entries

Perl scripts utilizing BioPerl modules were written to parse the BLASTX output produced in the EST2uni pipeline to determine the completeness of ESTs relative to matching Uniprot sequences (Table 3). Sequences were considered complete on their 5' or 3' end if the EST matched within ten amino acids of the corresponding end of the Uniprot entry.

#### Identification of microsatellites

The EST2uni pipeline identifies di-, tri- and tetra-nucleotide microsatellites using a modified version of Sputnik (C. Abajian, http://espressosoftware.com/sputnik/index.html. The minimum repeat unit length was set to 2 and the maximum repeat unit length to 4. The minimum microsatellite length was set to 24.

#### Functional Classification and Pathway Annotation

The Blast2GO [42] program was used to assign functional Gene Ontology (GO) annotations [43]. Assembled unigenes that had significant (e^-5^) BLASTX matches to the nr database were mapped to candidate GO terms. Annotation assignment is described in the Blast2GO paper and in the tutorial provided with the software. An e-value-hit-filter of 1.0e^-6^, an annotation cutoff of 55, a GO Weight of 5, and a Hsp-Hit Coverage Cutoff of 0 were used as Blast2GO annotation parameters.

Using the single-directional best hit (SBH) method as recommended by the KEGG Automatic Annotation Server (KAAS) [44] for sets of ESTs allowed us to assign unigene pathway annotations.

#### Mapping onto fish genome sequences

Assembled ESTs were mapped onto the genomes of tetraodon, medaka and stickleback using BLAT [45]. The BLAT manual suggests that for mapping ESTs to a genome across species one use a translated query against a translated database (-*q = dnax *and -*t = dnax*). The BLAT results were saved as blast tabular with comment lines (-*out = blast9*). The tabular blast output was then converted to a GFF3 formatted file using a slightly modified version of blast92gff3.pl (D. Gilbert, http://eugenes.org/gmod/genogrid/scripts/blast92gff3.pl). The GFF3 file is then loaded into a MySQL database, which can then be viewed as a track in GBrowse [46].

### Online access to the resource

Individual ESTs have been deposited with GenBank (Acc# GR588780-705678). The assembled contigs can be viewed through a variety of interfaces at http://www.bouillabase.org.

## Authors' contributions

BYL, HD, EP and FD constructed the various libraries. AEH prepared the libraries for sequencing. MC, JFB, KLC and TDK performed the bioinformatic analyses and prepared the manuscript. All authors read and approved the final paper.

## Supplementary Material

Additional file 1 **Figure S1: GO annotation**. Proportion of unigenes annotated with GO terms for each of the three GO functional categories (biological process, molecular function, and cellular component). Click here for file
