# Supplementary figures and images for: An EST resource for tilapia based on 17 normalized libraries and assembly of 116,899 sequence tags
Source: BMC Genomics. 2010 Apr 30;11:278. doi: 10.1186/1471-2164-11-278 (PMC2874815; doi:10.1186/1471-2164-11-278)

## biological\_process Level 2

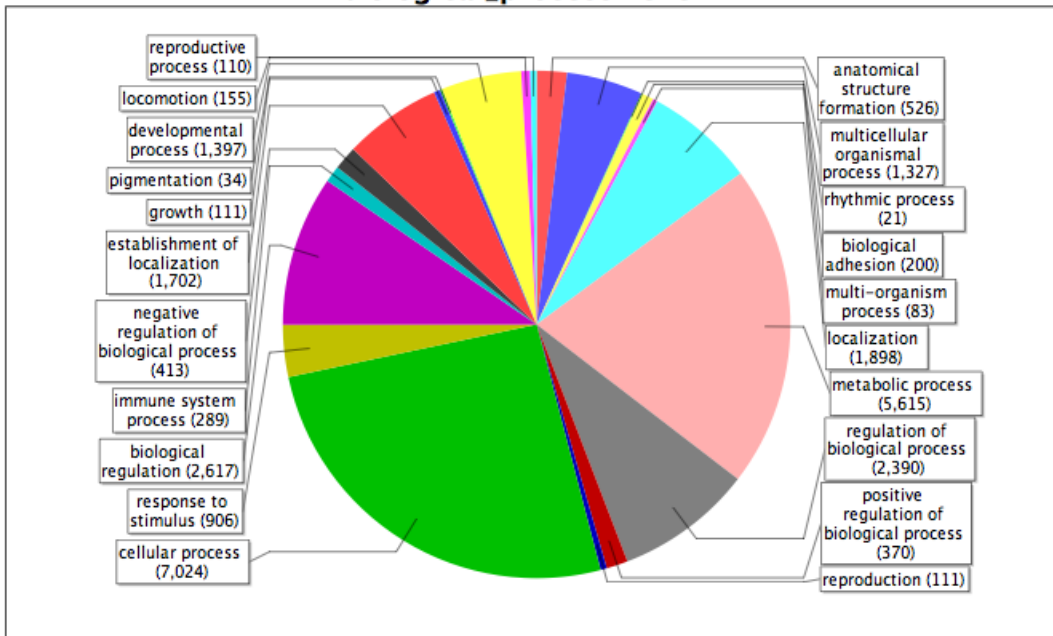

## cellular\_component Level 2

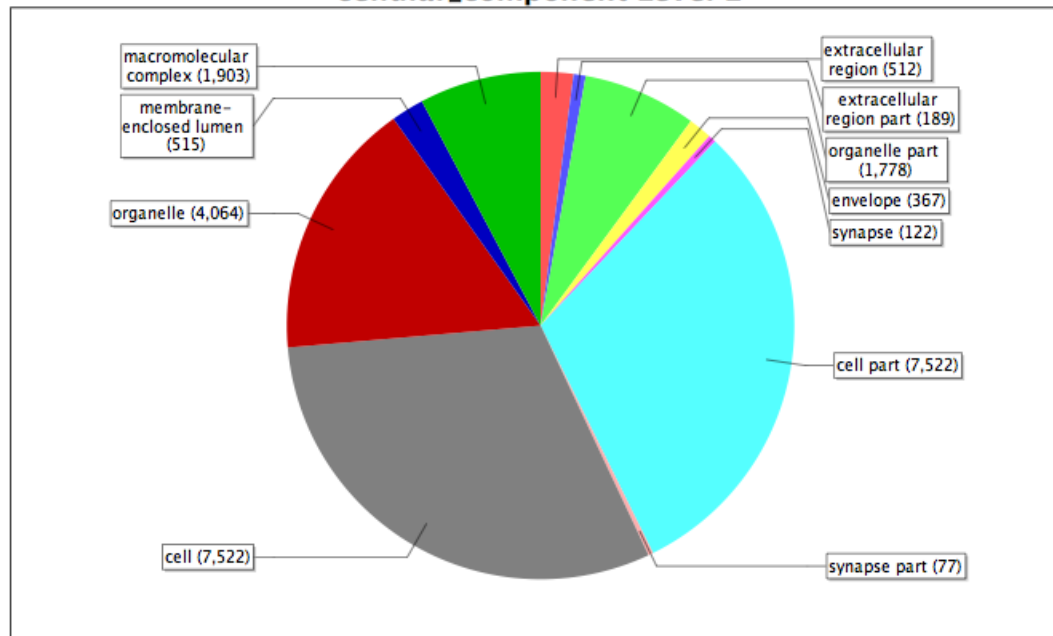

## molecular\_function Level 2

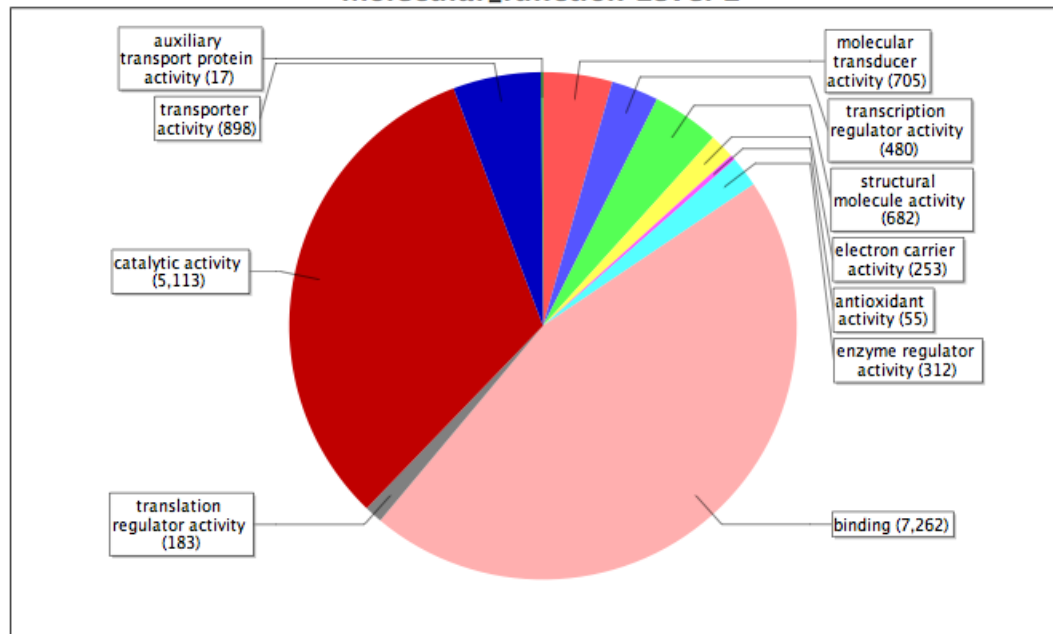

Supplement: Additional file 1 — Figure S1: GO annotation. Proportion of unigenes annotated with GO terms for each of the three GO functional categories (biological process, molecular function, and cellular component). [file 1471-2164-11-278-S1.PDF]
